# Gut microbiota: a potential target for traditional Chinese medicine intervention in coronary heart disease

**DOI:** 10.1186/s13020-021-00516-0

**Published:** 2021-10-22

**Authors:** Tian-Yi Cheng, Jia-Xin Li, Jing-Yi Chen, Pei-Ying Chen, Lin-Rui Ma, Gui-Lin Zhang, Pei-Yu Yan

**Affiliations:** Faculty of Chinese Medicine, State Key Laboratory of Quality Research in Chinese Medicines, Macau University of Science and Technology, Macau, People’s Republic of China

**Keywords:** Coronary heart disease, Gut microbiota, Traditional Chinese medicine

## Abstract

Coronary heart disease (CHD) is a common ischaemic heart disease whose pathological mechanism has not been fully elucidated. Single target drugs, such as antiplatelet aggregation, coronary artery dilation and lipid-lowering medicines, can relieve some symptoms clinically but cannot effectively prevent and treat CHD. Accumulating evidence has revealed that alterations in GM composition, diversity, and richness are associated with the risk of CHD. The metabolites of the gut microbiota (GM), including trimethylamine N-oxide (TMAO), short-chain fatty acids (SCFAs) and bile acids (BAs), affect human physiology by activating numerous signalling pathways. Due to the advantage of multiple components and multiple targets, traditional Chinese medicine (TCM) can intervene in CHD by regulating the composition of the GM, reducing TMAO, increasing SCFAs and other CHD interventions. We have searched PubMed, Web of science, Google Scholar Science Direct, and China National Knowledge Infrastructure (CNKI), with the use of the keywords “gut microbiota, gut flora, traditional Chinese medicine, herbal medicine, coronary heart disease”. This review investigated the relationship between GM and CHD, as well as the intervention of TCM in CHD and GM, and aims to provide valuable insights for the treatments of CHD by TCM.

## Background

Currently, cardiovascular disease (CVD) has become the leading cause of death globally. An estimated 17.9 million people died from CVD in 2019, representing 32% of all global deaths [1]. There are approximately 290 million CVD patients, of whom 11 million suffer from coronary heart disease (CHD) [2]. Hypertension, diabetes, dyslipidaemia, obesity, inflammation, smoking, alcohol consumption, insufficient fruit and vegetable intake, lack of physical activity, and high psychosocial pressure are the main risk factors for the occurrence and development of CHD [3, 4]. In recent years, novel drugs such as nicorandil, ivabradine, and trimetazidine have been developed. The combination of interventional therapy and drugs has been developed, including revascularization therapy and drug-eluting stents. These treatments can effectively relieve the symptoms of patients but can hardly prevent the progression of CHD. However, with advances in metagenome technology and ribonucleic acid sequencing technology metagenome technology, accumulating evidence has revealed that the gut microbiota (GM) is associated with CHD and its risk factors. It is estimated that modulating the GM will become an emerging therapeutic strategy for the prevention of CHD.

Because of its multitarget, multichannel and multicomponent synergistic action, traditional Chinese medicine (TCM) has been used to treat CHD for thousand years. Recent studies have found that TCM has outstanding curative effects on CHD by regulating the GM, which provides a new therapeutic target for the prevention and treatment of CHD in the future.

## Composition and metabolites of GM affect the occurrence of CHD

GM is a general term for microbes existing in the human gut, consisting of more than 1000 species of bacteria and 1 × 10^14^ communities [5], including Firmicutes, Actinobacteria, Bacteroidetes, Proteobacteria, Verrucomicrobia, etc. Among them, Firmicutes and Bacteroidetes are the dominant strains [6]. According to pathogenicity, GM is split into probiotics, opportunistic pathogens and pathogenic bacteria. The balance of probiotics and pathogeneticbacteria can protect the intestinal mucosal barrier, help the body intake nutrients, coordinate metabolism and immunity, and prevent pathogenic microorganisms from invading the body [7]. At the same time, GM can affect human health through its metabolites, such as trimethylamine N-oxide (TMAO), short-chain fatty acids (SCFAs) and bile acids (BAs). These metabolites act as signalling molecules to regulate metabolism and the inflammatory response in CHD patients [8].

### The composition of GM affects the occurrence of CHD

**Fig. 1:**
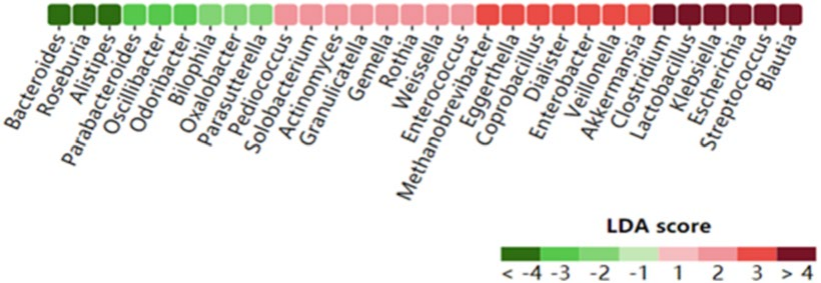
Linear discriminant analysis (LDA) coupled with coronary heart disease and gut microbiota. (Marker taxa with LDA < 0 are Health enriched, while those with LDA > 0 are cardiovascular diseases enriched.)

Aberrant compositional changes in GM are associated with the onset and progression of CHD. According to the Gmrepo database [9], it performed a metagenome-wide association study on stools from individuals with atherosclerotic cardiovascular disease and healthy controls (Fig. 1). A clinical study involving atherosclerotic patients (n = 218) and healthy people (n = 187) corroborated the approximate results. The study described a higher abundance of Enterobacteriaceae, *Solobacterium moorei*, and Enterobacter aerogenes along with *Atopobium parvulum*, Streptococcus spp. and Lactobacillus salivarius in atherosclerotic patients. In contrast, a relative decrease in Bacteroides, *Roseburia intestinalis*, Prevotella and Faecalibacterium cf. prausnitzii was detected in atherosclerotic patients [10]. Another comparative study conducted in CHD patients (n = 29) and 35 healthy volunteers revealed a significant increase in the proportion of Firmicutes and a decrease in Bacteroidetes [11]. This characteristic change was also identified in a clinical study in which Firmicutes was increased and Bacteroidetes was decreased in CHD patients [12]. Accordingly, it has been proposed that the Firmicutes/Bacteroidetes (F/B) ratio is considered a biomarker of gut dysbiosis and a diagnostic marker to identify CHD patients [13, 14]. Firmicutes can ferment intake food, while Bacteroidetes is responsible for absorbing and degrading polysaccharides [15, 16]. An animal experiment investigated that after transplanting obese germ-free mice with higher profusion of Firmicutes and nonobese mice with higher abundance of Bacteroides, the obese recipient had a higher dietary energy harvesting ability [17]. Increasing capacity for energy harvesting but poorer degradtion will result in higher intake of fat. In addition, Firmicutes was enriched due to high fat intake, generating a vicious cycle and leading to endotoxins and inflammation, while Bacteroidetes showed the opposite effect [18–20]. The beneficial function of Bacteroidetes in CHD was further authenticated in an animal experiment [21], which indicated that the increase in *Bacteroides vulgatus* and *Bacteroides dorei* could effectively decrease lipopolysaccharides and suppress proinflammatory immune responses.

More studies have demonstrated that alterations in GM could affect metabolic risk factors for CHD. Actinobacteria are considered to have a negative correlation with cholesterol. An increase in Actinobacteria reduced atherogenic lipid metabolites, proinflammatory cytokines and atherosclerotic lesions [22, 23]. *Prevotella copri* and Bifidobacteria positively ameliorated glucose tolerance, alleviating the development of CHD [24]^25^. Bifidobacteria play a beneficial role, giving the rein a reduction in AS. Eubacterium was positively connected with enhanced visceral fat mass in people and higher TMAO levels [26], while Bifidobacteria displayed an inverse relationship to Eubacterium, reducing lipid accumulation [21, 27]. Moreover, Tariq et al. indicated that hypercholesterolaemic patients (n = 15) had an increase in Proteobacteria. After treatment, hypercholesterolaemic patients had an increase in anti-inflammation-associated bacteria such as *Akkermansia muciniphila*, *Faecalibacterium prausnitzii*, and Oscillospira [28].

The theory of TCM holds that the pathogenesis of a disease lies in the struggle between Zheng qi (healthy qi) and Xie qi (pathogens). When pathogens hurt the human body, Zheng qi will rise vigorously to expell Xie qi, destroying the relative balance of Yin and Yang. In a previous study, probiotics coordinated metabolism and immunity, preventing pathogenic microorganisms from invading the human body, which resembled Zheng qi. To resist pathogens, the human body will mobilize more Zheng qi. This provides a potential mechanism for explaining the increase in some probiotics in patients. Intestinal microflora dysbiosis is the imbalance between probiotics and pathogenetic bacteria, which is very similar to the pathogenesis of a disease in TCM.

### Metabolites of GM affect the occurrence of CHD

#### Trimethylamine N-oxide (TMAO) affects the occurrence of CHD (Fig. 2)

**Fig. 2:**
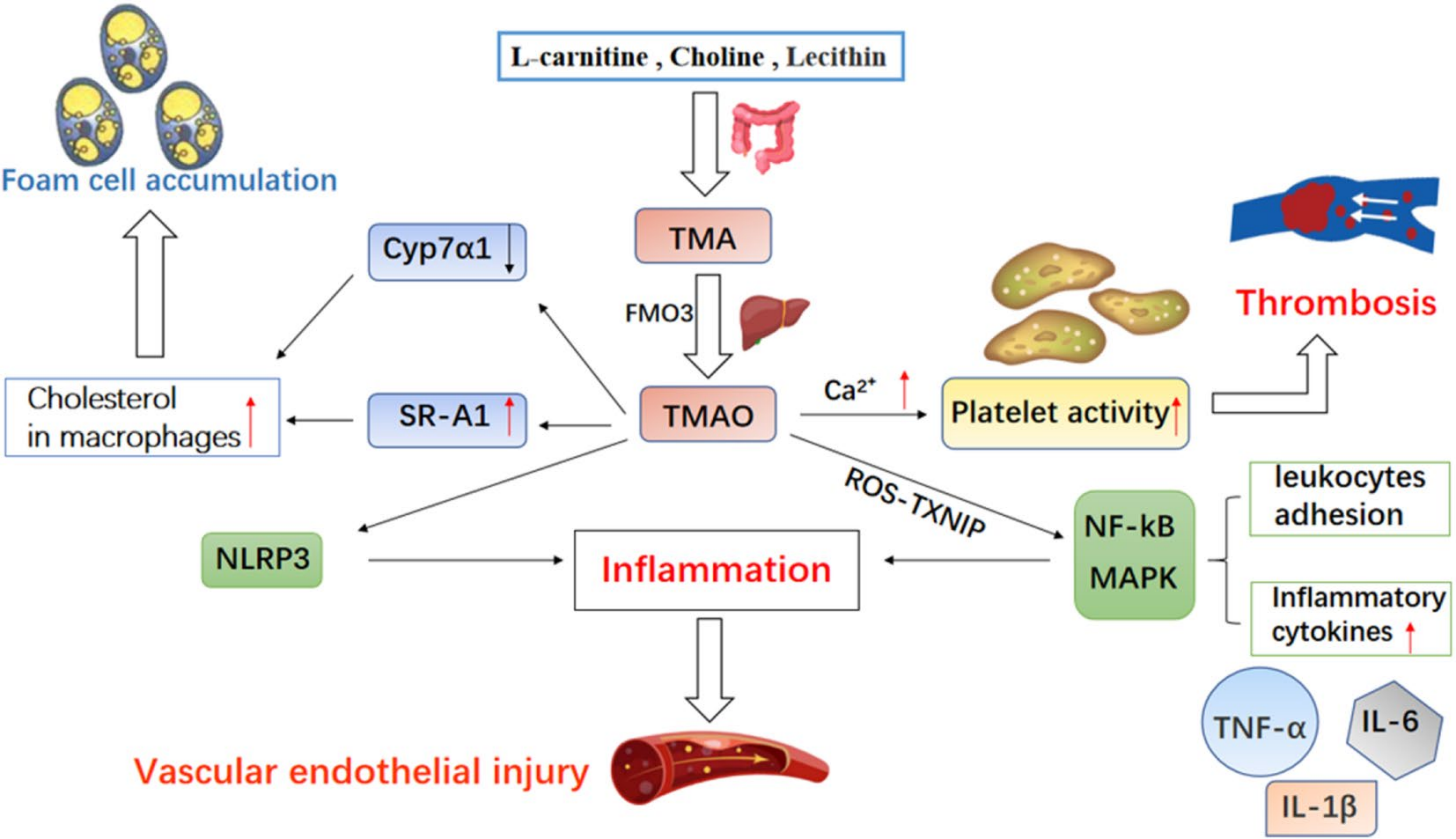
The mechanism of TMAO affecting CHD. *Cyp7a1* cholesterol 7α-hydroxylase, *SR-A1* scavenger receptor A1, *NLRP3* pyrin domain-containing-3, *TMA* trimethylamine, *FMO3* flavin-containing monooxygenase 3, *TMAO* trimethylamine N-oxide, *TXNIP* thioredoxin binding protein, *ROS* reactive oxygen species, *NF-kB* nuclear factor-kappa B, *MAPK* mitogen-activated protein kinase, *TNF-α* tumor necrosis factor-α, *IL-6* interleukin 6, *IL-1β* interleukin-1 beta

TMAO is a metabolite produced by choline and other substances dependent on the GM. According to Koeth’s research, the human GM metabolizes choline, phosphatidylcholine and l-carnitine to produce trimethylamine (TMA). TMA is further converted into TMAO catalysed by flavin monooxygenase (FMO) in the liver through oxidation [29, 30]. In addition to assistance in diagnosing and predicting CHD, TMAO also has the properties of causing AS and CHD. In a three-year follow-up study of 4007 patients with CHD, Tang et al. [31] found that elevated TMAO levels made a higher incidence of malignant cardiovascular events. TMAO has a better evaluation effect than traditional prognostic markers for cardiovascular events [32]. A prospective study of urban Chinese adults found that TMAO levels were still positively associated with CHD after adjusting for diet, blood lipids and other risk factors [33]. In an animal study, Apo E−/− mice transplanted with the C57BL/6 J strain (high TMAO production) showed elevated AS levels compared with mice treated with the NZW/Lac J strain (low TMAO production) [34].

At present, researchers explain the risk of CHD caused by TMAO through multiple mechanisms. First, TMAO causes vascular endothelial damage. A study showed that TMAO could activate the mitogen-activated protein kinase (MAPK) and nuclear factor-kappa B (NF-kB) signalling cascades of human aortic endothelial cells. Then, TMAO causes endothelial inflammation by releasing inflammatory cytokines and enhancing the adhesion of leukocytes to vascular endothelial cells [35]. When TMAO was administered to partially ligated the carotid artery in mice, the NLRP3 inflammasome in the intima of the blood vessels increased [36]. Sun et al. [37] suggested that the priming of NLRP3 was mediated through the ROS-TXNIP pathway. In the current study, TXNIP is a protein that connects ROS to the NLRP3 inflammasome. Subsequently, the SDHB/ROS pathway induced apoptosis of vascular endothelial cells and promoted AS in Apo E−/− mice [38]. Second, TMAO promotes thrombosis. Direct exposure of platelets to TMAO contributed to Ca^2+^ release from intracellular stores, which enhanced platelet hyperresponsiveness and promoted thrombosis [39]. Furthermore, TMAO promotes the deposition of cholesterol in macrophages and the formation of foam cells by upregulating the expression of scavenger receptor A (SRA) [40]. On the other hand, TMAO activated farnesoid X receptor (FXR) and small heterodimer partners to inhibit bile acid synthesis by reducing CYP7A1 expression [41]. This caused cholesterol accumulation and foam cell formation. Then, foam cells accumulate to form lipid streaks and even lipid plaques, which promote AS. This inferred that growing TMAO could accelerate the occurrence of AS. Thus, treatment regimens for CHD that reduce TMAO levels have become a hot spot.

#### Short-chain fatty acids (SCFAs) affect the occurrence of CHD (Fig. 3)

**Fig. 3:**
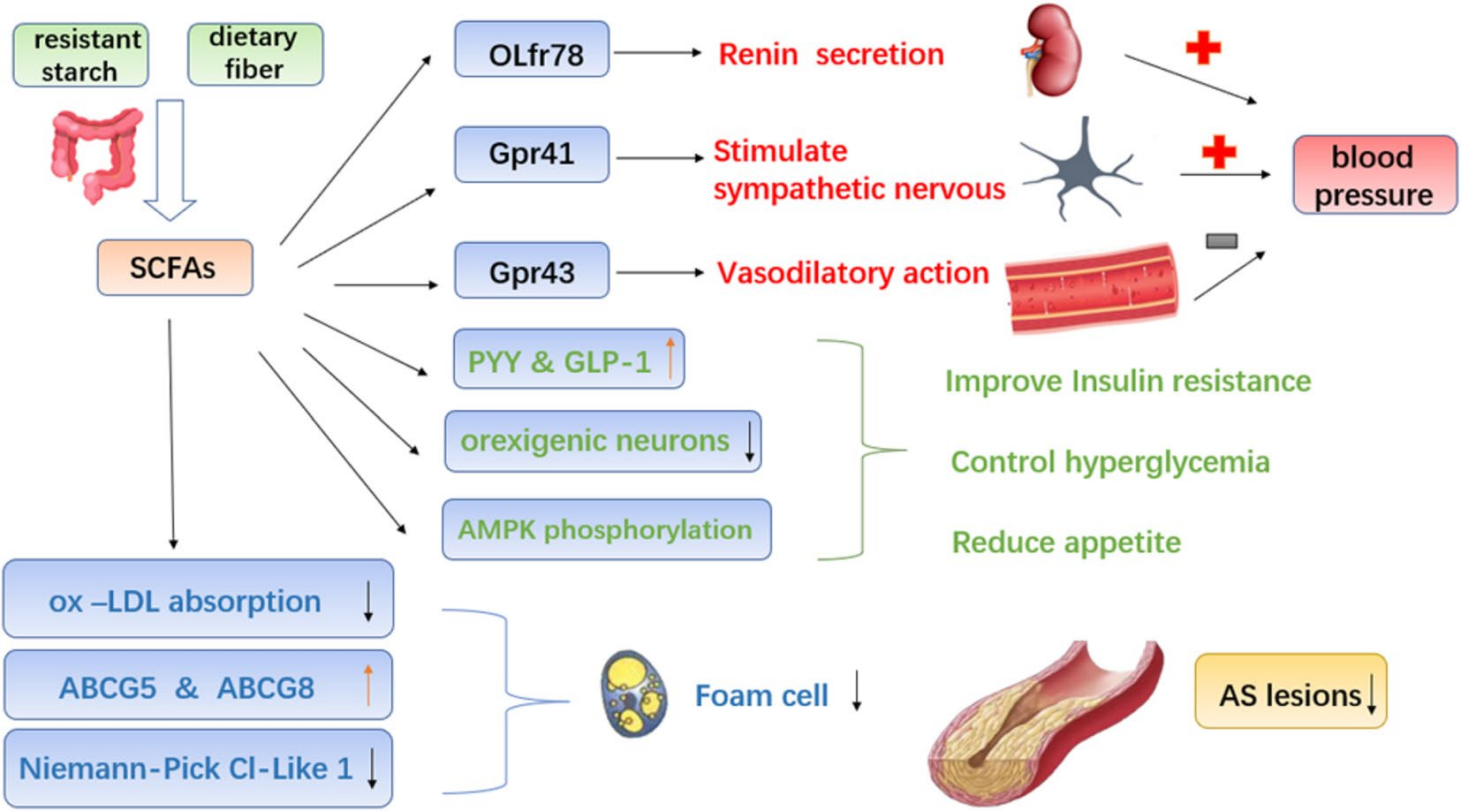
The mechanism of SCFAs affecting CHD. *SCFAs* short-chain fatty acids, *ox-LDL* oxidized low-density lipoprotein, *ABCG5* ATPbindingcassettetransportG5, *ABCG8* ATPbindingcassettetransportG8, *OLfr78* olfactory receptor 78, *Gpr41* G protein-coupled receptor 41, *Gpr43* G protein-coupled receptor 43, *PYY* peptide tyrosine tyrosine, *GLP-1* glucagon-like peptide-1, *AMPK* AMP-activated protein kinase, *AS* atherosclerosis

SCFAs are organic fatty acids with carbon atom numbers less than 6, mainly acetic acid, propionate, and butyrate [42], produced by digestion of resistant starch and dietary fibre in the intestine by different bacteria (Table 1). As a risk factor for CHD, hypertension can be regulated by SCFAs binding to olfaction receptor 78 (OLFR78), G-protein coupled receptor 41 (GPR41), and G-protein coupled receptor 43 (GPR43) [43]. Studies have shown that butyrate reduces cholesterol absorption by downregulating the expression of Niemann-pick C1-Like1 and upregulating the expression of ATP-binding cassette transporters G5 and G8, thereby further reducing the formation of AS [44]. Butyrate also reduced the expression of oxidized low-density lipoprotein (ox-LDL)-stimulated macrophages, which lessened ox-LDL absorption and the formation of foam cells [45]. In an animal study conducted by Bartolomaeus et al. [46], AS lesions were significantly reduced in apolipoprotein E knockout-deficient mice after receiving propionate. Furthermore, SCFAs could activate adenosine 5′-monophosphate (AMP)-activated protein kinase (AMPK) phosphorylation and glucagon-like peptide-1 (GLP-1) secretion to decrease inflammation and promote insulin resistance [47].Moreover, SCFAs via the gut-brain neural circuit suppressed the activity of orexigenic neurons that expressed neuropeptide Y in the hypothalamus to reduce appetite [48]. Ingestion of propionate significantly increased peptide YY and GLP-1, ultimately reducing food intake and improving insulin sensitivity [49]. It is now recognized that SCFAs are beneficial to reduce the formation of AS, but many experiments are based on cell and animal experiments, so further investigations are needed.

**Table 1:** SCFAs and their producers

SCFAs	Producers
Butyrate	*Eubacterium rectale*, *Roseburia intestinalis*, *Clostridium butyricum* [50], *Roseburia inulinivorans*, *Faecalibacterium prausnitzii* [51], *Coprococcus catus*, *Eubacterium ballii* [52]
Propionate	*Phascolarctobactreium succinatutens*, *Mycobacterium tuberculosis* [51], *E. col*i, *Bacteroides fragilis* *B. ruminicola* [53], *Salmonella typhimurium* [54], *Roseburia inulinivorans* [55], *Dalister* spp., *Veilonella* spp., *Megasphaera elsdenii*, *Coprpcoccus catus*, *Ruminocossus obeum* [52]
Acetate	Most of gut microbiome *Akkermansia mucinipbilia*, *Bateroides* spp., *Bifidobacterium* spp., *Prevotella* spp., *Chrostridium* spp., *Streptococcu*s spp. [52], *Roseburia* spp., *Coprococcus* spp., *Faecalibacterium prausnitzii*, *Roseburia intestinalis* [56], *Ruminococcus* spp., *Blautia drogentrophica* [57]

*spp.* species pluralis

#### Bile acids (BAs) affect the occurrence of CHD

BAs are important components of bile and play a fundamental role in fat metabolism [58]. The metabolism of BAs is also connected with plasma glucose [59] and CHD by a 20-year follow-up experiment [60]. Bile salt hydrolase (BSH), which exists in GM, can catalyse the hydrolysis of conjugated bile salts into deconjugated BAs, maintaining the balance of BA metabolism. In a recent study, BSH was shown to be produced by several intestinal bacteria, such as Clostridium [61], Lactobacillus [62], Bifidobacterium [63, 64], Bacteroides [65] and Enterococcus [66]. Song et al. investigated whether there was a prominent discrepancy between healthy people and patients with diabetes and atherosclerosis in terms of BSH [67]. Dysbiosis of the GM caused a reduction in BSH, which significantly impaired the metabolism of BAs and made cholesterol beyond the normal range as well as an inability to maintain glucose homeostasis, promoting the formation of AS [66].

In an animal experiment, Sayin et al. found that GM activated the expression of fibroblast growth factor 15 (FGF15) by reducing tauro-beta-muricholic acid (TβMCA) levels and activating FXR. The expression of FGF15 further inhibited CYP7A1, reducing the generation of BAs [68]. Interventions with probiotics [69] and Tempol [70] could downregulate the FXR/FGF15 pathway to influence the metabolism of fat. Collectively, targeting gut flora to inhibit the FXR/FGF15 pathway could be a treatment for AS.

## Mechanism of gut microbiota in the aetiology of TCM for CHD

Regarded as the foundation of digestion and absorption, the spleen and stomach in TCM theory are similar to the physiological function of the GM. The stomach governs the intake and digestion of the diet, while the spleen is responsible for transforming the decomposed diet into qi, blood and fluid (the material foundation of human activities). Eventually, these materials are transmitted to organs and other tissues by the spleen, offering adequate nourishment. Bacteria in the gut play analogous roles in the digestion and absorption of nutrients converted from food. Thus, it is believed that the GM belongs to the category of spleen and stomach in TCM.

The theory of TCM holds that the pathogenesis of CHD is characterized by phlegm, blood stasis and toxin. An improper diet, lack of rest, severe stress and other pathogenic factors will cause dysfunction of the spleen and stomach, mainly manifesting as qi deficiency. The deficiency of qi indicated that the power to promote fluid flow is insufficient, and stagnant fluid will accumulate to form phlegm. Excessive phlegm can be absorbed into the meridian, leading to blood stasis. The long-term blockade of phlegm and blood stasis in vessels cause the occurrence of heat toxin. Phlegm, blood stasis and toxin obstruct the heart meridian, becoming the pathological mechanism for cardiovascular diseases.

**Fig. 4:**
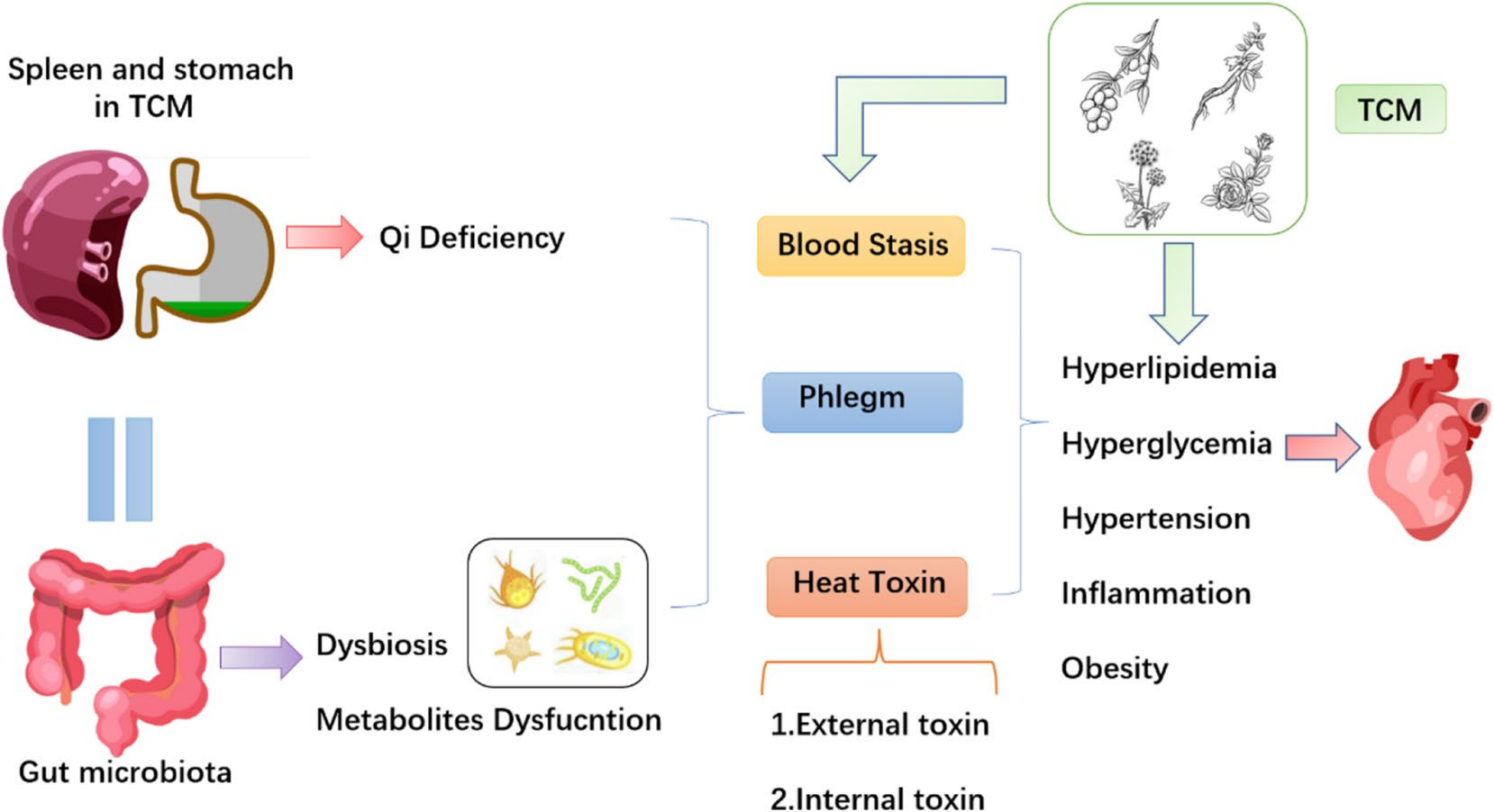
Gut microbiota linked to spleen and stomach in TCM and coronary heart disease. *TCM* traditional Chinese medicine

Dysfunction of the spleen and stomach closely resembles gut flora disorder. Blood lipids and TMAO are biomarkers of phlegm and stasis in TCM [71]. TMAO, converted from food by GM, not only causes hyperlipidaemia but also promotes thrombosis. Microbiota dysbiosis and an increase in pathogenic bacteria can be regarded as “external toxins”, while microbiota-derived metabolite dysfunction is regarded as an “internal toxin” [72], resulting in hypertension, hyperglycaemia, hyperlipidaemia, inflammation and obesity. This is the similar pathological basis of heart vessel occlusion, explaining the phlegm, blood stasis and toxin in TCM from a modern microbiology perspective. Therefore, TCM treats CHD by promoting blood circulation, removing phlegm and blood stasis, and detoxifying; on the one hand, TCM intervenes in CHD by reducing hypertension, hyperglycaemia, hyperlipidaemia, inflammation and obesity by adjusting the intestinal flora (Fig. 4).

## Therapeutic intervention with TCM

Most herbs are administered orally and absorbed through the intestine. Bacterial enzymes in the intestine are directly involved in the absorption and metabolism of TCM. Several investigations have shown that TCM monomers and compounds might improve the symptoms of CHD and its risk factors by modulating the gut flora [73]. This paper discussed Therapeutic Intervention with TCM according to classification of monomers, single herbs, herb pairs, decoctions.

### Monomers (Table 2)

**Table 2:** Herb monomers and gut microbiota

Monomers	Herbs	Physiological function related to gut microbiota	Gut microbiota	References
Resveratrol	*Polygonum cuspidatum*, *Ampelopsis japonica*, Smilax glabra Roxb	(1) Improve the dysbiosis of gut microbiota (2) Reduce TMA via inhibiting the metabolism of choline and attenuate TMAO-induced AS (3) Activate the BSH and promote the catabolism of BAs (4) Decrease mRNA expression of genes related to fatty acid synthesis, lipogenesis and adipogenesis through the FiaF signaling pathway (5) Improve glucose homeostasis in obese individuals (6) Lower the inflammatory state of obese individuals	*Increased:* the ratio of Bacteroides to Firmicutes, Lactobacillus, Bifidobacterium, Bacteroides, Parabacteroides *Decreased:* *Enterococcus faecalis*, Proteobacteria, Turicibacteraceae, Moryella, Lachnospiraceae,	Chen et al. [74] Qiao et al. [75] Sung et al. [76]
Berberine	*Coptis chinensis* Franch, cortex phellodendri	(1) Reduce atherosclerosis (2) Revert the high-fat diet-induced structural changes of gut microbiota and enrich SCFA-producing bacteria (2) Lower arterial and intestinal expression of proinflammatory chemokines and cytokines (3) Suppress anaerobic production of TMA and inhibit the choline-to-TMA transformation (4) Reduce body weight, blood glucose levels and intestinal inflammation	*Increased:* *Akkermansia* spp., Allobaculum, Butyricoccus, Blautia, Bacteriodes, Phascolarctobacterium, Ruminococcus, Coprococcu, Lactobacillus *Decreased:* *C. sporogenes*, *A. hydrogenalis*, Prevotella, Proteus	Zhu et al. [77] Li et al. [78] Zhang et al. [79] Zhang et al. [80]

*TMA* trimethylamine, *TMAO* trimethylamine N-oxide, *AS* atherosclerosis, *BSH* bile salt hydrolase, *BAs* bile acids, *spp* species pluralis, *SCFAs* short chain fatty acids

#### Resveratrol

Resveratrol (RSV), a natural polyphenolic compound present in many medicinal herbs (including *Polygonum Cuspidatum*, *Ampelopsis japonica* and Smilax glabra Roxb), has the clinical effect of improving blood glucose and lipid homeostasis [81], reducing fat mass and blood pressure [82], and alleviating oxidative stress and inflammation [83]. A growing body of evidence supports the hypothesis that RSV plays a role primarily through remodelling the gut microbiota. A study suggested that RSV attenuated TMAO-induced AS by increasing the ratio of Bacteroides to Firmicutes (B/F), thereby reducing TMA by inhibiting the metabolism of choline [74]. In addition, significantly increasing the abundance of Lactobacillus and Bifidobacterium, RSV activated BSH and promoted the catabolism of BAs in the intestine to regulate the synthesis of BAs in the liver [74]. In terms of obesity, RSV improved the dysbiosis of gut microbiota induced by the high-fat diet, including increasing the ratio of B/F and the growth of Lactobacillus and Bifidobacterium (negatively correlated with body weight) and inhibiting the growth of Enterococcus faecalis (positively correlated with body weight). The FiaF signalling pathway significantly decreased the mRNA expression of genes related to fatty acid synthesis, lipogenesis and adipogenesis [75]. A group [76] carried out a faecal microbiota transplant (FMT) and showed that decreases in Proteobacteria with resveratrol-FMT were associated with changes in sphingomyelins and phosphatidylcholines. They have been implicated in inflammation and the inflammatory pathway [84], thereby lowering the inflammatory state of obese mice. In addition to the changes in gut microbiota induced by resveratrol, the composition of the gut microbiome regulates the production of resveratrol metabolites (including Piceid [85], dihydroresveratrol and 3,4′-dihydroxy-trans-stilbene [86]), whose concentrations in humans after ingestion are higher than those of their parent molecules and can have similar biological effects.

#### Berberine

Berberine (BBR), an isoquinoline alkaloid, can be found in several medicinal herbs, including *Coptis chinensis* Franch and cortex phellodendri. BBR and BBR-containing herbs have been shown to be effective and safe in antiatherosclerotic [87], antilipidaemic [88], anti-inflammatory [89] and antiobesity [90] applications. Recent studies have shown that the gut microbiota is one of the connections between the poor oral bioavailability of BBR and its pharmacological effects. The gut microbiota converts BBR into its absorbable form of dihydroberberine (dhBBR), whose absorption rate is fivefold higher than that of BBR in animals. DhBBR is unstable in solution and can then be reoxidized to BBR in intestinal tissues [91]. Zhu et al. [77] investigated the association between alterations in the gut microbial structure and the antiatherosclerotic effect of BBR in HFD-fed ApoE−/− mice. BBR treatment increased *Akkermansia* spp. abundance markedly, contributing to the lower arterial and intestinal expression of proinflammatory chemokines and cytokines as well as the reduction of atherosclerosis. The results by Li et al. [78] from in vitro, ex vivo to in vivo studies proved that BBR could suppress anaerobic production of TMA in both bacterial isolates and the complex gut microbial community, especially showing stronger inhibition of the choline-to-TMA transformation in the detected strains *A. hydrogenali*s and *C. sporogenes*. It may be the main potential mechanism of BBR in reducing TMA production, ultimate TMAO formation and aortic plaque area. Moreover, BBR showed reverting effects on HFD-induced structural changes in the gut microbiota [79], including the enrichment of SCFA-producing bacteria and a reduction in microbial diversity. Those belonging to putative SCFA-producing bacteria, including Allobaculum, Butyricoccus, Blautia, Bacteroides and Phascolarctobacterium, were significantly increased by BBR to resist obesity-related metabolic disorders. By modifying the gut microbiome, BBR reduced body weight, blood glucose levels and intestinal inflammation in db/db mice [80]. Changes in the gut microbiome were characterized by an increased relative abundance of SCFA-producing bacteria (e.g., Ruminococcus, Coprococcu, Butyricimonas) and other probiotics, including Lactobacillus and Akkermansia. A decreased relative abundance of opportunistic pathogens (e.g., Prevotella, Proteus) was also observed.

### Herbs (Table 3)

**Table 3:** Herbs and gut microbiota

Herbs	Components	Physiological function related to gut microbiota	Gut microbiota	References
Mulberry leaves	Mulberry leaf water extracts	(1) Promote SCFAs-produced gut microbial fermentation and the excretion of fecal sterol and bile acid (2) Reduce the serum total cholesterol level and the atherosclerotic index (3) Modify the disturbed gut microbiota to be restored (4) Improve lipid metabolism and prevent body fat accumulation	*Increased:* Leptotrichia, Bacteroidetes *Decreased:* Cyanobacteria Proteobacteria	He et al. [92] Ma et al. [93]
	Mulberry leaf flavonoids, polysaccharides and alkaloids	(1) Generate SCFAs (2) Regulate blood glucose level	*Increased:* Bacteroides *Decreased:* Desulfovibrio, Roseburia, Lachnospiraceae	Wang et al. [94] Zhang et al. [95] [96]
	Mulberry dietary fiber and polyphenols	Reduce weight	*Increased:* N/A *Decreased:* Lachnespiraceae, Clostridiales	Li et al. [97]
*Astragalus membranaceus*	Astragalus Polysaccharides	(1) Restore the balance of gut microbiota (2) Reduce the energy intake from HFD and the weight growth rate	*Increased:* the ratio of Bacteroides to Firmicutes, *Decreased:* Proteobacteria	He et al. [96]
	Astragali Radix vesicle-like nanoparticles	(1) Improve the gut microbiota dysbiosis (2) Reduce the fasting blood glucose and improve insulin resistance	*Increased:* the ratio of Bacteroides to Firmicutes, Muribaculaceae *Decreased:* Proteobacteria Lactobacillaceae Lachnospiraceae	Gao et al. [98]
	Calycosin	Balance harmful and beneficial gut microbiota	*Increased:* Bifidobacterium lactis, Lactobacillus mesenteroides *Decreased***:** Pathogenic bacteria, Enterococcus hirsutum, Enterobacter mesenteroides	Zhang. [99]
	Astragaloside IV	(1) Up-regulate the expression of AMPK/SIRTI and PI3K/AKT proteins to alleviate injuries from insulin resistance and oxidative stress (2) Up-regulate abundance of SCFAs-producing bacteria (3) Lower blood glucose and maintain body weight	*Increased:* Alistipes, Odoribactercan, Riken, Parabacteroides, Akkermansia *Decreased:* Lachnespiraceae, Clostridiales, **Proteobacteria**	Xiao et al. [100] Meng et al. [101]
*Ganoderma lucidum*	*Ganoderma lucidum* polysaccharides	(1) Regulate gut microbiota of type 2 diabetic (2) Promote the production of SCFAs (3) Affect some pathways through gut microbiota, to alleviate the symptoms	*Increased:* Bifidobacterium, Blautia, Clostricibacter, Coprococcus *Decreased:* Leuconostoc, Enterococcu, Dorea	Ding [102]
	*Ganoderma lucidum* spore oligosaccharides	(1) Regulate structure of the gut microbiota (2) Promote the production of SCFAs	*Increased:* Prevotella, Lactobacillus, Bifidobacterium, Faecalibacterium *Decreased:* Escherichia coli	Yang et al. [103]
	*Ganoderma lucidum* mycelium	(1) Alleviate gut microbiota of obesity (2) Maintain intestinal barrier integrity and reduce metabolic endotoxemia (3) Reduce and improve insulin resistance inflammation	*Increased:* the ratio of Bacteroides to Firmicutes, *Decreased:* Escherichia spp.	Chang et al. [104]
Ginseng	Ginsenoside Rg5	(1) Improve gut microbiota of Diabetic (2) Repair the intestinal barrier and alleviates metabolic endotoxemia related inflammation (3) Improve insulin resistance and blood glucose in diabetic	*Increased:* the ratio of Bacteroides to Firmicutes, Clostridium clusters XIVa, XVIII, and IV, Bacteroidetes, Proteobacteria, Faecalibacterium, Dialist *Decreased:* Dorea, Escherichia Shigella	Wei et al. [105] Yue 2021 [106]
Seaweed	Seaweed polysaccharides	(1) Regulate structure of the gut microbiota (2) Promote the production of SCFAs (3) improve the symptoms of diabetic (4) change the secretion and metabolism of mucin in intestinal mucus and against some infection (5) Increase Carbohydrate-Active enzymes and anti-obesity	*Increased:* the ratio of Bacteroides to Firmicutes, Clostridium cluster XIVa, Parabacteroides and Bacteroides *Decreased:* Clostridium cluster XIVb and XI	Chen et al. [107] Cheng et al. [108] Deville et al. [109] Nguyen et al. [110]
	Insoluble dietary fiber	(1) Regulate structure of the gut microbiota (2) Promote the production of SCFAs and improve the blood glucose level and fat metabolism	*Increased:* the ratio of Bacteroides to Firmicutes, Verrucomicrobia, Akkermansia *Decreased:* N/A	Zhang et al. [111]
Rhubarb	Rhubarb anthraquinone	(1) Regulate structure of the gut microbiota (2) Enhance intestinal barrier and reduce LPS induced inflammation (3) Promote the production of SCFAs and induce GLP-1 secretion to ameliorate insulin resistance (4) Anti- obesity	*Increased:* the ratio of Bacteroides to Firmicutes, Clostridium**,** Lactobacillus, Akkermansia, Roseburia *Decreased:* Desulfovibrio	Yu et al. [112] Regnier et al. [113] Cui et al. [114] Wang et al. [115]
*Salvia miltiorrhiza*	Salvianolic acid B	(1) Regulate structure of the gut microbiota (2) Improve insulin sensitivity and lipid metabolism disorder (3) Alleviate gut permeability	*Increased:* Bifidobacterium**,** Adlercreutzia, Lactobacillales *Decreased:* Bacteria genera Helicobacter, Desulfovibrio, Mucispirillum, Gram-negative Proteobacteria, Deferribacteres	Lin et al. [116] Zhou et al. [117]

*SCFAs* short chain fatty acids, *N/A* not available, *HFD* high-fat-diet, *AMPK* AMP-activated protein kinase, *SIRTI* silent information regulator 1, *PI3K* phosphatidylinositol 3-kinase, *AKT* V-akt murine thymoma viral oncogene homolog, *GLP-1* glucagon-like peptide-1, *LPS* lipopolysaccharide

#### Mulberry leaf

Mulberry leaves have been used as traditional medicine, and their traditional effects include dispersing wind and heat, purging heat from the liver and improving eyesight, cooling blood and haemostasis. Its main ingredients, such as mulberry flavonoids, mulberry polysaccharides, mulberry fibre, mulberry polyphenols and mulberry alkaloids, have been reported to play roles in its antihyperglycaemic and antilipidaemic effects by modulating the gut microbiota. Mulberry leaf water extracts (MWEs) could promote SCFA-produced gut microbial fermentation and the excretion of faecal sterol and bile acid, thus helping reduce the serum total cholesterol level and the atherosclerotic index in HFD-fed mice [92]. Similarly, Wang et al. [94] described that mulberry polysaccharides might promote the growth of Bacteroides. In turn, *Bacteroides ovatus* and *Bacteroides cellulosilyticus* could degrade mulberry polysaccharides into monosaccharides and oligosaccharide fragments and generate SCFAs that are beneficial to intestinal health. Ma et al. [93] revealed that MWE modified the disturbed gut microbiota to be restored in obese rats (including an increased abundance of Leptotrichia and Bacteroidetes and decreased abundance of Cyanobacteria and Proteobacteria), which may be a mechanism of MWE in improving lipid metabolism and preventing body fat accumulation. In addition, Li et al. [97] reported the synergistic interaction between mulberry dietary fibre and polyphenols in anti-obesity by regulating gut microflora for the first time. Their molecular interactions affect the bioavailability and beneficial effects in the food matrix [118]. Mulberry dietary fibre and polyphenols regulate the Firmicutes content to a normal level and reduce the amount of Lachnespiraceae (belonging to Firmicutes/Clostridiales) to achieve weight loss. A group [96] studied the effects of mulberry leaf flavonoids, polysaccharides and alkaloids on gut microflora in db/db diabetic mice. After administration, Desulfovibrio, Roseburia, Lachnospiraceae and Bacteroidetes were markedly regulated from disorder, which effectively regulated blood glucose levels, especially in the alkaloid group. This is consistent with the hypoglycaemic mechanism of alkaloids, that is, reducing postprandial hyperglycaemia by inhibiting α-glucosidase in the small intestine [119].

#### Astragalus membranaceus

*Astragalus membranaceus* is a commonly used Qi-tonics medicine in the clinical practice of traditional Chinese medicine. Flavonoids, polysaccharides and saponins, the main ingredients of Astragali membranaceus, have functions of regulating immunity, lowering blood glucose and improving cardiac function. In the relevant mechanism of their effectiveness, gut microflora plays an essential role in improving their bioavailability and has impacts on their efficacy [120]. A study in HFD-induced obese mice [96] showed that Astragalus polysaccharides (APS) restored the balance of gut microbiota by increasing the relative abundance of Bacteroidetes and Firmicutes and reducing the abundance of Proteobacteria bacteria. The transplantation of gut microbiota in APS-fed mice could significantly reduce the weight growth rate of HFD-fed mice. This effect of gut microbiota could only be played under the condition of HFD, which showed that the gut microbiota changed by APS reduce the energy intake from HDF. Gao et al. [98] reported that after 3 weeks of administration, compared with the model group, Astragali Radix vesicle-like nanoparticle (VLN) groups could markedly reduce the fasting blood glucose of db/db diabetic mice by improving gut microbiota dysbiosis (an increased relative abundance of beneficial bacteria and the ratio of B/F). Moreover, calycosin is a flavonoid component of Astragalus. Zhang [99] suggested that calycosin may regulate intestinal health by balancing harmful and beneficial gut microbiota. It inhibited the growth of pathogenic bacteria, Enterococcus hirsutum and Enterobacter mesenteroides in a dose-dependent manner and, at suitable concentrations, promoted the growth of beneficial bacteria such as Bifidobacterium lactis and Lactobacillus mesenteroides. Astragaloside IV (AST) is one of the major active components of astragalosides and has no first pass effect after oral administration [121]. Xiao [100] demonstrated that AST increased genera such as Alistipes, Odoribactercan, and Riken in db/db mice, which could upregulate the expression of AMPK/SIRTI and PI3K/AKT proteins to alleviate injuries from insulin resistance and oxidative stress. Meanwhile, the upregulated abundance of SCFA-producing bacteria, including Blautia, Rikenellaceae, Alistipes, Lachnospiraceae, and Butyrivibrio, was observed. Furthermore, Meng et al. [101] explored the hypoglycaemic mechanism of astragaloside IV combined with berberine (BBR). They reported that the combination of astragaloside IV and BBR was more effective for lowering blood glucose and maintaining body weight than alone in type 2 diabetic rats. In the combined group with a decrease in blood glucose, the abundances of Parabacteroides and Akkermansia were increased, while the abundance of Proteobacteria was reduced.

#### Ganoderma lucidum

*Ganoderma lucidum* is a traditional Chinese medicine made of dried fruiting bodies of fungi that has the function of Tonifying Qi and tranquilizing the mind, stopping cough, and relieving asthma. Recent studies have shown that *Ganoderma lucidum* has antioxidant, hypoglycaemic, and hypolipidaemic effects and has potential therapeutic effects on AS, whose mechanism is related to gut microbiota. Ding's research shows that [102]. *Ganoderma lucidum* polysaccharides promote the production of SCFAs through the fermentation of intestinal microflora, such as acetic acid, propionic acid, and butyric acid, which regulate the blood glucose level and impair glucose tolerance in diabetic rats. Meanwhile, *Ganoderma lucidum* polysaccharide can significantly change the composition and structure of gut microbiota to promote the growth of beneficial bacteria, such as Bifidobacterium, Clostribacteriaceae, Blautia, and Coprocccus, and inhibit the growth of harmful bacteria, such as Dorea and Leuconostoc. It affects the activity of various pathways through gut microbiota, such as bacterial chemotaxis, flagellar assembly, and glycan biosynthesis and metabolism, which can alleviate the symptoms of type 2 diabetic rats. Additionally, *Ganoderma lucidum* spore oligosaccharides extracted from *Ganoderma lucidum* spore powder can promote the production of SCFAs and increase the abundance of beneficial bacteria such as Lactobacillus and Prevotella [103]. In addition, Chang [104] and his colleagues found that *Ganoderma lucidum* mycelium (WEGL) can improve the intestinal barrier to prevent LPS of gram-negative bacteria from entering enterohepatic circulation, activate TLR4 signalling and reduce macrophage infiltration, and enhance Treg accumulation in liver and adipose tissue, which can reduce inflammation in HFD-fed mice. Phosphorylation of serine 307 on IRS-1 was inhibited by WEGL in hepatic and adipose tissues to improve insulin resistance. In terms of obesity, WEGL can reduce the F/B ratio and Escherichia spp. in HFD-fed mice and increase a variety of bacterial species negatively related to obesity.

#### Ginsenoside

Ginsenoside is the main active component of ginseng, which can not only alleviate the occurrence and development of AS but also improve its risk factors. Ginsenoside Rb1 can alleviate AS by reducing hyperlipemia [122], inhibiting inflammation [123], and inhibiting calcification of vascular smooth muscle cells (VSMCs) [124]. According to the experimental results of Yange Wei [105], ginsenoside Rg5 can significantly reduce the ratio of F/B and increase Clostridium clusters XIVa, XVIII, and IV in db/db mice. Clostridium clusters XIVa, XVIII, and IV have been shown to reduce inflammation by activating Treg cells [125]. Yue's experiment found that protopanaxatriol saponins could increase the abundance of Faecalibacterium and Dialist and reduce the abundance of Dorea and Escherichia Shigella [106]. In addition, Rg5 can improve the expression of occludin and ZO-1 to repair the intestinal barrier. By regulating the composition of the gut microbiota, it also significantly reduces LPS levels and inhibits the TLR4-related inflammatory signalling pathway to alleviate metabolic endotoxaemia-related inflammation. In addition, Rg5 can reverse the JNK pathway and activate NF-kB, which improves insulin resistance and blood glucose in diabetic db/db mice [125].

#### Seaweed

Seaweed is rich in dietary fibre and divided into soluble dietary fibre and insoluble dietary fibre. According to the American Association of Cereal Chemists (AACC), dietary fibre includes polysaccharides, oligosaccharides, lignin, and associated plant substances that promote beneficial physiological effects, such as the attenuation of blood cholesterol and blood glucose [126]. Recent studies have shown that saccharides in seaweed can effectively treat and prevent coronary atherosclerosis from many aspects [127–129]. Seaweed polysaccharides can reduce the F/B ratio and improve the symptoms of diabetic mice [107, 108]. In Deville’s study [109], seaweed polysaccharide increases SCFAs in the intestine and reduces pH in the intestine, which is conducive to inhibiting the growth of harmful bacteria. Additionally, it changes the secretion and metabolism of mucin in intestinal mucus to influence the adherence and translocation of gut microbiota across the epithelial wall and against some infections [110]. In anti-obesity diets, seaweed polysaccharide-supplemented diets increase carbohydrate-active enzymes (CAZy), which include an increase in some enzymes associated with a decline in the human body mass index, by increasing bacteria that digest dietary polysaccharides and decreasing potentially pathogenic bacteria. In addition, Zhang's experiment [111] showed that insoluble dietary fibre in seaweed could dose-dependently reduce the number of Firmicutes and increase Verrucomicrobia in HFD mice. At the same time, by adjusting the proportion of akkermansia and a. muciniphila, the SFCA content in the intestine of HFD mice was affected to further improve the blood glucose level and fat metabolism of HFD mice.

#### Rhubarb

Rhubarb is one of the most ancient and common herbs in Chinese medicine, and the first record can be traced back to Shen Nong’s herbal classic. The main chemical compositions of rhubarb include anthraquinones, anthrones, stilbenes, tannins, polysaccharides, etc. [130]. Traditional Chinese medicine believes that rhubarb has the functions of removing accumulation with purgation, clearing heat-fire, clearing heat and toxins from the blood, dredging meridian and relieving blood stasis, promoting diuresis and anti-icteri. Anthraquinone of rhubarb is the main component of rhubarb, which is mainly absorbed in the intestine and has antioxidant [131] and anti-inflammatory [132] functions, regulating lipid metabolism [133]. Yu and her colleagues [112] found that rhubarb anthraquinone had stronger antibacterial activity against pathogenic bacteria than probiotics through in vitro culture. Rhubarb extract can increase the mRNA expression of REG3 and PLA2g2 in the colon and induce the mRNA expression of intestinal epithelial cell turnover protein in high fat and high sucrose diet rats [113]. Additionally, high-doserhubarb anthraquinone-glycoside treatment can significantly increase the expression of ZO-1 and occludin in type 2 diabetes mellitus rats [114]. These results indicate that rhubarb can enhance intestinal barrier integrity by increasing the expression of specific antimicrobial peptides and major tight junction proteins in the intestine and promoting epithelial cell renewal, which reduces LPS-induced inflammation. In terms of the structure of the gut microbiota, Wang's [115] experiments showed that rhein in rhubarb anthraquinone can increase the B/F ratio to reduce obesity and improve glucose metabolism in diabetic mice. In an experiment by Cui et al. [114], both rhubarb anthraquinone-glycoside and metformin reduced pathogenic bacteria such as Desulfovibrio and increased probiotic bacteria (Lactobacillus, actobacillus, and akkermansia). In addition, his study demonstrated that rhubarb anthraquinone-glycoside increases the abundance of some probiotics (Clostridium and Lactobacillus) and SCFA-producing bacteria (akkermansia and Roseburia). These bacteria provide energy for intestinal L cells to secrete GLP-1 or directly induce GLP-1 secretion to stimulate insulin secretion and improve the sensitivity of peripheral tissue to insulin to ameliorate insulin resistance.

#### Salvia miltiorrhiza

*Salvia miltiorrhiza* is a commonly used drug for promoting blood circulation and removing blood stasis. It is widely used in the treatment of various cardiovascular diseases and has the effects of promoting blood circulation and removing blood stasis, dredging meridians and relieving pain, clearing heart fires and removing annoyance, cooling blood and eliminating carbuncles [134]. Studies have shown that salvianolic acid A in *Salvia miltiorrhiza* can inhibit the oxidation of LDL, inhibit inflammation, and improve endothelial function to ameliorate AS [135, 136]. Salvianolic acid B (Sal B) can reverse gram-negative bacteria in HFD mice, which is positively associated with LPS elevation and increases the abundance of adlercreutzia to improve gut permeability [116]. By maintaining intestinal motility, *Salvia miltiorrhiza* could improve the tolerance of intestinal mucosal epithelial cells to ischaemia and hypoxia to protect intestinal barrier function [117]. Meanwhile, *Salvia miltiorrhiza* could enhance the barrier function of diabetic mice through the expression of tight junction proteins in the intestine [137]. Thus, *Salvia miltiorrhiza* can reduce metabolic endotoxaemia by reducing LPS and protecting the intestinal barrier. Furthermore, in Lin's study [116], salvianolic acid B inhibited the LPS/TLR4 signalling pathway by inhibiting the abundance of gram-negative bacteria and improving insulin sensitivity and lipid metabolism disorder in HFD mice.

### Herb pair and Decoctions (Table 4)

**Table 4:** Herb pair/prescription and gut microbiota

Herb pair/prescription	Herbs	Physiological function related to gut microbiota	Gut microbiota	References
Paired-drugs of *Astragalus membranaceus* and *Salvia miltiorrhiza*(HD)	*Astragalus membranaceus*, *Salvia miltiorrhiza*	(1) Improve insulin resistance and hyperlipidemia (2) Reduce the level of circulating inflammatory factors, fat content and endotoxemia (3) Regulate blood pressure	*Increased:* The ratio of Bacteroidetes to Firmicutes Lactobacillus intestinalis, Akkermansia, *Akkermansia muciniphila* *Decreased:* N/A	Han et al. [138]
Xiexin Tang (XXT)	Rhei Rhizoma, Scutellariae Radix, Coptidis Rhizoma	(1) Regulate structure of the gut microbiota (2) Ameliorate hyperglycemia, lipid metabolism dysfunction and inflammation (3) Exhibit vasorelaxant andantihypertensive effects	*Increased:* Lactobacillus, Blautia, Adlercreutzia *Decreased:* Alloprevotella, Papillibacter, Prevotellaceae NK3B31, Lachnospiraceae UCG-001	Xiao et al. [139] Wei et al. [140] Wu et al. [141]
Bu Yang Huan Wu Decoction (BYHWD)	*Angelica sinensis*, *Astragalus membranaceus*, *Amygdalus persica*, *Carthamus tinctorius*, *Paeonia lactiflora*, *Ligusticum chuanxiong*, *Pheretima aspergillum*	(1) Regulate structure of the gut microbiota (2) Reduce TMAO levels and reducing LDL-C (3) Affect the plasma metabolites profile of ischemic symptoms (4) Antioxidation and reduce the hypercoagulable state of blood	*Increased:* Bacteroidetes *Decreased:* Escherichia coli, Clostridium, Dsulfovibrionaceae, Coriobacteriacea, Rikenellaceae, Peptococcaceae	Li et al. [142] Sun et al. [143] Wu et al. [144]

*TMAO* trimethylamine N-oxide, *N/A* not available, *LDL-C* lipoprotein cholesterol

#### Herb pair of *Astragalus membranaceus* and *Salvia miltiorrhiza*

Herb pair, a common form of compatibility in the clinical prescription of TCM, is a pair of two relatively fixed medicines that have a certain theoretical basis and combination rules [145]. *Astragalus membranaceus* is known as "the best of all drugs for tonifying Qi", and *Salvia miltiorrhiza* has been proven to. Promote blood circulation and remove blood stasis. Accordingly, the herb pair of *Astragalus membranaceus* and *Salvia miltiorrhiza* (HD) is based on the theory of Tonifying Qi and activating blood circulation [146] and has been used to treat CVDs for many years. Han et al. [138] explored the effects and possible mechanism of HD on gut microbiota in spontaneously hypertensive rats (SHRs). In this study, an increased F/B ratio and abundance of Lactobacillus intestinalis, Akkermansia, and *Akkermansia muciniphila* were observed after administration of HD, which improved insulin resistance and hyperlipidemia and reduced the levels of circulating inflammatory factors, fat content and endotoxemia. Therefore, HD might regulate blood pressure and treat CHD by regulating related factors.

#### Xiexin Decoction

Xiexin Decoction (XXD) is a TCM formula recorded in the masterpiece Synopsis of the Golden Chamber written by ZhongjingZhang. XXD consists of Rhei Rhizoma, Scutellariae Radix, and Coptidis Rhizoma, which has the effect of Clearing heat, detoxifying and drying dampness. Some clinical and animal trials have reproduced the beneficial effects of XXD on AS. XXD effectively regulated lipid metabolism by decreasing triglycerides and LDL and increasing HDL. XXD also downregulated the expression of inflammation-related proteins, such as intercellular adhesion molecule-1 (ICAM-1) and vascular cell adhesion molecule-1 (VCAM-1), playing an anti-inflammatory role. In addition, XXD reduced the apoptosis of vascular endothelial cells induced by ox-LDL, which was related to inhibiting Caspase-9 and Caspase-3 [147]. A recent study showed that intragastric administration of XXD for 4 weeks increased GM-derived SCFA-producing ability by improving key SCFA synthetic enzymes, such as acetate CoA transferase (BUT) and acetate kinase (ACK).The results demonstrated that XXD could effectively ameliorate lipid metabolism disturbance and alleviate inflammation [139]. In a comparitive clinical study, treatment with XXD combined with conventional Western medicine (n = 60) significantly reduced blood glucose and blood lipid levels by increasing probiotics [148]. Wei et al. reported that rats treated with XXD exhibited changes in SCFA production and anti-inflammatory bacteria such as Alloprevotella, Adlercreutzia, Barnesiella, and Blautia, ameliorating hyperglycaemia, lipid metabolism dysfunction and inflammation [140]. Furthermore, XXD could increase Lactobacillus abundance, exhibiting vasorelaxant and antihypertensive effects [141].

#### Bu Yang Huan Wu Decoction

Bu Yang Huan Wu Decoction (BYHWD) is a classical prescripetion of TCM recorded in the Correction on Errors in Medical Classics, initiated by Qingren Wang, emphasizing the importance of tonifying Qi, promoting blood circulation and removing blood stasis. BYHWD consists of *Angelica sinensis*, *Astragalus membranaceus*, *Amygdalus persica*, *Carthamus tinctorius*, *Paeonia lactiflora*, *Ligusticum chuanxiong* and *Pheretima aspergillum*. It has been used clinically to treat ischaemic stroke and CHD [149–151]. As a significant risk factor for AS, homocysteine (Hcy) could induce apoptosis of vascular endothelial cells by increasing the production of reactive oxygen species (ROS) [152]. However, BYHWD inhibited the NF-kB-dependent pathway to decrease ROS and Hcy, further preventing [153] Meta-analysis also showed that BYHWD was effective in CHD, reducing TC and TG levels [154].

BYHWD treatment (7.37 g/kg/d) for 4 weeks reduced TMAO levels and the abundance of Escherichia coli and Clostridium, reduced LDL-C and ameliorated cardiovascular performance [142, 143]. It also significantly downregulated Dsulfovibrionaceae, Coriobacteriaceae, Rikenellaceae and Peptococcaceae, which affected the plasma metabolite profile of ischaemic symptoms, mainly threonine, tyrosine, arginine and other amino acid metabolism. In addition, BYHWD upregulated oleamide with antioxidant effects and warfarin to reduce the hypercoagulable state of blood [144]. Therefore, this provides a new provement for improving the ischaemia mechanism of BYHWD.

In accordance with the theory of TCM, the above herbs can be concluded to be qi-invigorating herbs, heat-clearing and detoxicating herbs and blood circulation-promoting and blood stasis-removing herbs. Qi-invigorating herbs not only improve the function of the spleen and stomach but also promote blood circulation in the pulse, which fundamentally solves the generation of pathological products, such as phlegm and blood stasis. Blood circulation-promoting and blood stasis-removing herbs ensure blood vessel unobstructed, preventing stagnation from turning into heat toxin. With a cold nature, heat-clearing and detoxicating herbs reduce the damage of internal and external toxins to the body. These three kinds of drugs are in line with our abovementioned TCM treatment principles for CHD, which can be used by clinicians to choose appropriate drugs to intervene in CHD [155].

In addition, according to the theory of viscera syndrome differentiation, 28.79% of CHD disease locations are in the spleen and stomach [156]. Most herbal medicines are administered orally and absorbed through the intestine. The GM is directly involved in the absorption and metabolism of TCM. Consequently, GM is closely related to the spleen and stomach. The fact that the abovementioned herbal medicines mostly have channel tropism of the spleen and stomach also confirms this theory. Therefore, the mechanism by which TCM intervenes in CHD by regulating the GM can provide a theoretical basis for TCM "treating coronary heart disease from spleen and stomach", which has clinical guiding value.

## Conclusions

Undoubtedly, there are trillions of bacteria in the human intestine, and the gut microbiota is considered the "second human genome”. Currently, an unhealthy lifestyle, abuse of antibiotics and intestinal environment disorders may lead to gut dysbiosis. Previous studies have shown that dysbiosis of the gut microbiota and changes in metabolites, such as TMAO, SCFAs and BAs, are associated with the occurrence and development of coronary heart disease, hypertension, hyperglycaemia, hyperlipidaemia and obesity.

Thus, methods of treating CHD by regulating the GM are emerging. Depending on the multitarget preponderances, TCM can markedly promote probiotic abundance, reduce TMAO, increase SCFAs and supply appropriate BAs to intervene in CHD. These findings extend previous observations and support the nation that TCM may be a new therapeutic target for the prevention and intervention of CHD.

Because CHD is increasingly appearing among young individuals, the prevention of CHD has become increasingly significant. By understanding the relationship between the patient gut microbiota and CHD treatments, doctors can prevent CHD. Doctors can use TCM to help people who are susceptible to CHD by regulating the GM. We can also combine a mixed preparation of probiotics with TCM to prevent CHD by promoting the GM, which can eventually reduce the occurrence as well as the societal and economic costs of CHD.

## Data Availability

Wu, S.C.; Sun, C.Q.; Li, Y.Z.; Wang, T.; Jia, L.H.; Lai, S.Y.; Yang, Y.L.; Luo, P.Y.; Dai, D.; Yang, Y.Q., et al. GMrepo: a database of curated and consistently annotated human gut metagenomes. Nucleic Acids Research 2020, 48, D545-D553, https://doi.org/10.1093/nar/gkz764.

## Abbreviations

CHD: Coronary heart disease
GM: Gut microbiota
TCM: Traditional Chinese medicine
TMAO: Trimethylamine N-oxide
SCFAs: Short chain fatty acids
BAs: Bile acids
CNKI: China National Knowledge Infrastructure
CVD: Cardiovascular disease
F/B: Firmicutes/Bacteroidetes
Apo E−/− mice: Apolipoprotein E knockout (ApoE−/−) mice
AS: Atherosclerosis
ox-LDL: Oxidized low-density lipoprotein
AMPK: AMP activated protein kinase
Spp: Species pluralis
BSH: Bile salt hydrolase
RSV: Resveratrol
B/F: Bacteroidetes/Firmicutes
FMT: Faecal microbiota transplant
BBR: Berberine
HFD: High-fat-diet
db/db mice: Diabetes mice
MWEs: Mulberry leaf water extracts
APS: Astragalus polysaccharides
VLN: Vesicle-like nanoparticle
AST: Astragaloside IV
WEGL: *Ganoderma lucidum* Mycelium
VSMCs: Vascular smooth muscle cells
AACC: American Association of Cereal Chemists
CAZy: Carbohydrate-active enzymes
Sal B: Salvianolic acid B
N/A: Not available
HD: The herb pair of *Astragalus membranaceus* and *Salvia miltiorrhiza*
SHRs: Spontaneously hypertensive rats
XXD: Xiexin Decoction
BYHWD: Bu Yang Huan Wu Decoction
TG: Triglyceride
VLDL: Very low-density lipoprotein
HDL: High-density lipoprotein
LDL: Low-density lipoprotein
